# Respiratory system responses to a maximal apnoea

**DOI:** 10.1113/EP091346

**Published:** 2024-11-21

**Authors:** Colin D. Hubbard, Troy J. Cross, Garrett Z. Merdich, Dario Vrdoljak, Nikola Foretic, Željko Dujić, Joseph W. Duke

**Affiliations:** ^1^ Department of Biological Sciences Northern Arizona University Flagstaff Arizona USA; ^2^ Heat and Health Research Centre, Sydney School of Health Sciences, Faculty of Medicine and Health The University of Sydney Sydney NSW Australia; ^3^ Faculty of Kinesiology University of Split Split Croatia; ^4^ Department of Integrative Physiology University of Split School of Medicine Split Croatia

**Keywords:** apnoea divers, breath‐hold divers, fatigue, involuntary breathing movements, respiratory muscles

## Abstract

A maximal apnoea provides significant challenges to one's physiological systems, including significantly altered arterial blood gases, and requires a highly integrative response from multiple systems, that is, changes in blood pressure, maintenance of cerebral blood flow, etc. Previous work and reviews have focused on the cardiovascular responses to a maximal apnoea, but very little work has focused upon the responses of the respiratory muscles and respiratory mechanics. This is important because of the changes to arterial blood gases leading to an increased drive to breath and the appearance of involuntary respiratory muscle contractions. This review outlines what is known about how the respiratory system responds to a maximal apnoea. We put forth the hypothesis that the respiratory muscles may become fatigued following a maximal apnoea and that the respiratory muscles of elite divers may be more fatigue‐resistant, which could be an important feature of these individuals which allows them to be successful in this sport. Finally, we provide direction for future work to explore the long‐term health of apnoea diving.

## Abstract (highlights)

- **What is the topic of this review?**
  This narrative review outlines the known and unknown responses of the respiratory system to a maximal apnoea.
- **What advances does it highlight?**
  This review enhances our understanding of the respiratory responses to a maximal apnoea by outlining how the respiratory muscles respond and how respiratory mechanics may change during or as a result of a maximal apnoea. We put forth novel hypotheses about these parameters, that is, development of respiratory muscle fatigue, and provide future directions for experimental studies using apnoea as a physiological stressor.

## 1 INTRODUCTION

Apnoea diving, that is, freediving, is an activity that is performed for hunting and gathering, recreation, and for sport. As a hunting and gathering activity, apnoea diving has a long history dating back as far as 10,000 years ago (Quilter & Arriaza, 1997). Specifically, there is evidence that the Chinchorro, inhabitants of the Pacific coast of South America, had a diet consisting predominantly of food originating from the sea (Quilter & Arriaza, 1997). Likewise, there is fossil evidence that ancient people living near the coast of the Baltic Sea practiced freediving for seafood. The Ama have practiced apnoea diving for pearls and seafood off the coast of Japan and Korea for at least the past 2000 years (Rahn & Yokohama, 1965). However, apnoea diving as a sport is a somewhat recent activity having seen significant emergence over the last ∼30 years. At present, apnoea diving as a sporting discipline includes a variety of classifications depending upon whether it is performed under static or dynamic conditions, the number/type of fins used while swimming, and/or the type of descension device (if any). Accordingly, the duration of maximal breath‐hold is highly dependent upon the specific discipline being used.

At present, the world record, per the International Association for the Development of Apnea (AIDA), for a static apnoea is 11:35 min for men and 9:07 for women and the maximal distance dynamic apnoea with fins (monofin) is 301 m for men and 290 m for women (bi‐fin) (AIDA, 2019). Additionally, the record for maximal depth in the ‘no limit’ discipline is 214 m for men and 160 m for women (AIDA, 2019). Accordingly, these physical feats place a tremendous stress on the human body and require robust physiological responses to allow these athletes to attain such achievements. There are several excellent reviews on the physiology of apnoea diving (see Bain et al., 2018; Dujic & Breskovic, 2012; Lindholm & Lundgren, 2009; Patrician, Dujić et al., 2021), which have outlined the integrative responses to a maximal static apnoea. However, these reviews focused primarily on the cardiovascular system with minimal discussion of the responses of and stresses placed upon the respiratory system. In this review, we aim to outline the responses of the respiratory muscles and changes in respiratory mechanics to a maximal static apnoea performed at the water surface or dry, as well as make mention of the chronic impact of ‘apnoea training’. As part of this, we will discuss changes in arterial blood gases and how the drive to breathe changes, as they pertain to the respiratory muscles.

## 2 PHYSIOLOGY OF A BREATH‐HOLD

The ability of an individual to maintain a breath‐hold is dependent upon a multitude of factors. Relevant to the respiratory system, these factors include the amount of O_2_ stored in the lungs at the start of the apnoea, conservation of O_2_, and the amount of CO_2_ that is produced via metabolism. The onset of the physiological breakpoint, that is, end of the easy‐going phase (explained further below), is likely a multiplicative relationship between the aforementioned parameters and their impact on chemoreceptor sensitivity and output (Bain et al., 2018). Factors determining the termination of the maximal apnoea are less clear, but may be related to ‘respiratory variability’ and cerebral blood flow (Cross, Kavanagh et al., 2013), as well as ability to tolerate hypoxaemia and hypercapnia. Regardless, a maximal breath‐hold is separated, roughly in half, between the easy‐going and struggle phases.

**FIGURE 1:**
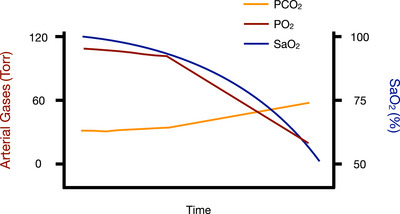
Changes in arterial blood gases during a maximal apnoea. Data are adapted from Bain et al. (2018) and Willie et al. (2014) where the orange line represents changes in arterial $P_{CO_{2}}$, the maroon line represents changes in arterial $P_{O_{2}}$, and the blue line represents changes in arterial O_2_ saturation ($S_{aO_{2}}$) over the course of a maximal apnoea. Note the severe hypoxaemia and hypercapnia present at the end of a maximal apnoea.

The easy‐going phase is a period of quiescence. The glottis is closed, and respiratory neuromuscular output is voluntarily inhibited. During this phase, the elite apnoea diver finds the breath‐hold to be effortless to maintain. Arterial $P_{O_{2}}$ is maintained at a relatively constant level (∼100 Torr) and $S_{aO_{2}}$ is normal (>95%) (Bain et al., 2016; Willie et al., 2014). Additionally, arterial $P_{CO_{2}}$ remains relatively unchanged compared to pre‐apnoea values, that is, 30 Torr (Figure 1). The mammalian diving reflex, during immersion, is profound and elicits an increase in mean arterial blood pressure (Breskovic et al., 2011; Ferrigno et al., 1997; Perini et al., 2008) and decrease in heart rate (Asmussen & Kristiansson, 1968; Ferrigno et al., 1997; Schagatay, 2009; Schagatay & Lodin‐Sundström, 2014), presumably, in an effort to conserve O_2_ and minimize the increase in $P_{CO_{2}}$. As the breath‐hold progresses there is a decline in arterial $P_{O_{2}}$ and $S_{aO_{2}}$ and an increase in arterial $P_{CO_{2}}$ (Bain et al., 2016; Willie et al., 2014). The increase in arterial $P_{CO_{2}}$ plays a critical role in determining the duration of the easy‐going phase of the breath‐hold. As $P_{CO_{2}}$ rises, the urge to breathe progressively increases and can no longer be, physiologically, ignored. This results in involuntary contractions of the respiratory muscles, that is, involuntary breathing movements (IBMs). However, the diver still has voluntary control over their glottis and so IBMs are made against a closed glottis. The appearance of IBMs marks the physiological breakpoint and the transition into the struggle phase. The exact threshold for the first IBM and the physiological breakpoint have not been fully elucidated yet, but are presumed to be $P_{CO_{2}}$ and $P_{O_{2}}$ dependent (Lin et al., 1974). Previous work demonstrated that the threshold for the onset of IBMs is a $P_{CO_{2}}$ of ∼48 ± 4 Torr (Breskovic et al., 2012), while the $P_{O_{2}}$ threshold is unknown. Some suggest that the physiological breakpoint is the result of acutely increased peripheral chemoreceptor stimulation due to the decreasing $P_{O_{2}}$ and increasing $P_{CO_{2}}$ (Lugliani et al., 1971; Parkes, 2006; Wasserman et al., 1975). In addition to arterial blood gases, it has been hypothesized that the breakpoint is the result of an increased accumulation of an excitatory state stemming from a cessation of respiratory movement (Godfrey & Campbell, 1968).

The struggle phase, as the name implies, is marked by a greater challenge to maintain the effort associated with the apnoea. The glottis remains closed, but respiratory neuromotor drive begins to increase profoundly as arterial $P_{O_{2}}$ decreases and arterial $P_{CO_{2}}$ increases. As a result, the activity of both peripheral and central chemoreceptors increases, which enhances the drive to breathe (Duffin, 2010; Mateika & Ellythy, 2003). Collectively, the enhanced chemoreceptor activation and the urge to breathe triggers the onset of IBMs. Support for this comes from Bain and colleagues, who demonstrated that the onset of IBMs occurred later in the apnoea when there was chemoreflex inhibition via dopamine (Bain et al., 2015). These respiratory muscle contractions, although not generating airflow, cause swings in intrathoracic and abdominal pressures. The frequency and amplitude of the IBMs (and swings in pressure) increase progressively in a ‘crescendoing’ pattern for the duration of the breath‐hold (Andersson & Schagatay, 1997; Cross, Breskovic et al., 2013; Dujic et al., 2009; Lin et al., 1974; McBride & Whitelaw, 1981). The struggle phase continues until the individual takes a breath. In the presence of intense IBMs during the struggle phase, splenic volume is further reduced, and mean arterial pressure transiently increases via renormalized inferior caval flow, which increases right atrial filling and stroke volume (Palada et al., 2008). The increased mean arterial pressure (and cardiac output) results in an increase in cerebral blood flow (Cross, Kavanagh et al., 2014; Dujic et al., 2009) allowing for cerebral O_2_ delivery to be largely maintained (Willie et al., 2014). Thus, IBMs have important haemodynamic consequences as well, likely facilitating the use of the last oxygen reserves before apnoea cessation.

Collectively, the changes in arterial $P_{O_{2}}$ and $P_{CO_{2}}$, in addition to lung and chest wall stretch, impact other physiological reflexes such as increasing sympathetic nervous system activation (Bain et al., 2018) and/or input from pulmonary stretch receptors and respiratory muscle spindle afferents (Whitelaw et al., 1981). The reduction in arterial $P_{O_{2}}$ and increased $P_{CO_{2}}$ during the breath‐hold causes marked vasoconstriction in the peripheral tissues, presumably to redirect oxygenated blood to critical organs (Baković et al., 2003; Ferretti, 2001; Kyhl et al., 2016; Mijacika, Frestad et al., 2017). Likewise, increased arterial $P_{CO_{2}}$ vasodilates both the carotid arteries and jugular veins as a mechanism to maintain cerebral O_2_ and CO_2_ transport (Bain et al., 2015, 2018; Cross, Breskovic et al., 2013; Stembridge et al., 2017; Willie et al., 2014). At the end of a maximal dry, surface apnoea, arterial $P_{O_{2}}$ has been observed to be as low as 20 Torr, $S_{aO_{2}}$ as low as 40–50% and arterial $P_{CO_{2}}$ as high as 60 Torr (Bain et al., 2016) (Figure 1; Willie et al., 2014). In essence, it is the changes in these parameters that ultimately cause the maximal apnoea to end, but more work is needed to confirm or refute this hypothesis.

As noted above, a maximal apnoea elicits a decline in arterial $P_{O_{2}}$ and an increase in arterial $P_{CO_{2}}$. Thus, a breath‐hold is maximized when arterial $P_{O_{2}}$ is as high as possible and arterial $P_{CO_{2}}$ is as low as possible at the beginning of the apnoea. In regard to arterial $P_{CO_{2}}$, the relationship between alveolar ventilation and alveolar/arterial $P_{CO_{2}}$ is such that a mild hyperventilation prior to a maximal apnoea would reduce arterial $P_{CO_{2}}$ and presumably increases maximal apnoea time. Importantly, it is vital not to reduce arterial $P_{CO_{2}}$ too much, that is, below ∼20 Torr, because cerebral blood flow is restricted and the possibility for blackout may increase (Pearn et al., 2015).

The role of O_2_, particularly O_2_ delivery to critical organs, in breath‐hold duration is crucial. The duration of a breath‐hold is increased when ‘stored’ O_2_ is the greatest (Schagatay, 2009). This notion is supported by the difference in apnoea duration with and without hyperoxia. The maximum static apnoea without hyperoxia is 11:35 and 9:07 for men and women, respectively. However, the world records, per *Guinness World Records*, are significantly longer, that is, 24:37 and 18:32 min in men and women, respectively, when divers are allowed to breathe 100% O_2_ before a maximal apnoea. Even without 100% O_2_ the elite apnoea diver has numerous tools at their disposal to maximize the amount of O_2_ that is ‘stored’ in the lungs and/or blood. First, a maximum apnoea is typically performed at or near total lung capacity, that is, maximum volume of gas in the lungs; and some divers may even practice glossopharyngeal insufflation (the act of ‘pumping’ and pressurizing the air in the lungs) to increase this storage further (Schagatay, 2009). Moreover, it is generally accepted that elite apnoea divers have larger lung volumes even when matched with controls (Carey et al., 1956; Ferretti & Costa, 2003). Some of this could be attributed to the apnoea training where divers practice glossopharyngeal insufflation and exsufflation in an attempt to reduce their residual volume and increase their vital capacity (Ferretti & Costa, 2003; Schagatay & Lodin‐Sundström, 2014; Schagatay et al., 2012). Vital capacities have been shown to be, on average, 1.8 L larger than non‐diver controls (Schagatay & Lodin‐Sundström, 2014). Others have shown practicing glossopharyngeal insufflation can result in an increase in total lung capacity up to 20% (Chung et al., 2010; Lindholm & Nyrén, 2005; Loring et al., 2007; Mijacika, Kyhl et al., 2017; Patrician, Spajić et al., 2021; Walterspacher et al., 2011). This is beneficial in a diver because larger lung volumes mean they can store a greater amount of oxygen, and thus maximal apnoea duration is longer (Breskovic et al., 2012; Schagatay, 2009). However, excessive glossopharyngeal insufflation can mechanically constrict the heart and could promote syncope (Batinic et al., 2011; Eichinger et al., 2010; Schipke et al., 2015).

## 3 RESPIRATORY MUSCLES AND MECHANICS

The primary muscles for inspiration are the diaphragm, external intercostals and scalenes. At rest, the diaphragm is responsible for ∼60% of the total pressure generated (De Troyer & Boriek, 2013). The pressure generated by the inspiratory muscles must be sufficient to overcome both lung elastic recoil and airway resistance. Once breathing is significantly elevated above resting ventilation, that is, one inhales close to total lung capacity, then the accessory muscles of inspiration are recruited (De Troyer & Boriek, 2013). These muscles include the sternocleidomastoid and scalenes. The energetic demand of the muscles of inspiration is, roughly, in proportion to the ventilatory demand (Milic‐Emili & Orzalesi, 1998). The primary muscles for expiration are the rectus abdominus and external obliques. Under resting conditions, the kinetic energy stored in the lungs during inspirations is, in general, sufficient to create a positive alveolar pressure and exhalation. However, once ventilation increases above resting levels and the individual expires below their functional residual capacity, then recruitment of the expiratory muscles, as well as the accessory muscles of expiration, that is, internal intercostals, is required to overcome the outward recoil of the chest wall.

During inspiration, the diaphragm contracts and descends, the thorax expands, the lungs are pulled downward (De Troyer & Wilson, 2016), and pleural pressure (*P*_PL_) decreases below atmospheric pressure and air flows into the lungs. Simultaneously, abdominal volume decreases and pressure in the abdomen (*P*_AB_) increases, as does pressure generated by the diaphragm (*P*_DI_). The diaphragm relaxes, as reflected by a decrease in *P*_DI_, thoracic volume decreases slightly, *P*_PL_ increases and air flows out of the lungs. Likewise, the expiratory muscles contract, abdominal volume increases and *P*_AB_ increases. The magnitude of the changes in the respiratory pressures is dependent upon the effort, that is, contractile strength, and the lung volume at which the inspiration/expiration occurs due to the compliance of the chest wall and lungs (see below).

Accordingly, an elite diver performing a maximal apnoea experiences increased respiratory muscle activity and repetitive loading during the struggle phase over the course of their competition and training schedule. This could lead to elite apnoea divers having stronger than normal respiratory muscles (Cross, Breskovic et al., 2013) and/or fatigue‐resistant respiratory muscles. Respiratory muscle strength can be quantified using voluntary manoeuvres, that is, maximal inspiratory and expiratory pressure manoeuvres (MIP, MEP), or involuntarily by stimulating the phrenic nerves (to quantify diaphragm strength; Bellemare & Bigland‐Ritchie, 1984; Johnson et al., 1993) or the thoracic root of the spinal cord (to quantify expiratory muscle strength; Kyroussis et al., 1996; Taylor & Romer, 2008, 2009). Yet, the assumption that divers have stronger respiratory muscles has not fully been determined. One study found maximal inspiratory pressures (MIP) in divers to be ∼145 cmH_2_O, which was comparable to non‐diver controls (∼132 cmH_2_O) (Tetzlaff et al., 2008).

**FIGURE 2:**
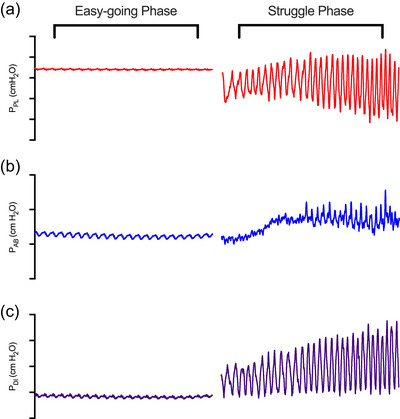
Example tracing of respiratory muscle pressures during a maximal apnoea. Changes in pleural pressure (*P*_PL_; a), abdominal pressure (*P*_AB_; b), and diaphragm pressure (*P*_DI_; c) during a maximal apnoea. Figure adapted from Cross, Breskovic et al. (2013).

In general, an apnoea diver performing a maximal apnoea will do so at total lung capacity, which requires the diaphragm and accessory muscles of inspiration to generate relatively large, albeit transient, pressures, that is, *P*_DI_ can frequently reach +50 to +80 cmH_2_O during a maximal inhalation (Cross, Breskovic et al., 2013). Importantly, this is probably lower than the maximal pressure‐generation ability of the diaphragm at functional residual capacity (+80 to +100 cmH_2_O) (Taylor & Romer, 2009). Then, once total lung capacity is attained, the glottis is closed and the apnoea is initiated. During the easy‐going phase, the individual is relaxing against the closed glottis and respiratory muscle are minimally, if at all, tonically active (Cross, Breskovic et al., 2013). Figure 2 displays a typical tracing of respiratory pressures during a maximal breath‐hold.

As noted above, the respiratory muscles are relatively inactive during the easy‐going phase, and tension, if any, is unchanging. However, once the physiological breakpoint is reached and the struggle phase begins, this is not the case (Cross, Breskovic et al., 2013). During this phase of the maximal apnoea the swings, that is, from end‐expiratory to end‐inspiratory lung volume, in *P*_DI_ are approximately +30 to +50 cmH_2_O near the end of a maximal apnoea (Cross, Breskovic et al., 2013). This change in *P*_DI_ during IBMs, although not resulting in airflow, is comparable to what is observed during dynamic exercise in an individual breathing at a ventilation of ∼100 L/min (Molgat‐Seon et al., 2018). Previous work has suggested that IBMs were performed primarily via inspiratory muscles (Agostoni, 1963), with others suggesting they were the result of both inspiratory and expiratory muscle activity (Kulik, 1964; Sempik, 1978; Sempik & Patrick, 1981). Recent work, however, has demonstrated progressive recruitment of expiratory muscles and internal and external intercostals during the struggle phase and that they contribute to swings in *P*_PL_ and *P*_AB_ during IBMs (Cross, Breskovic et al., 2013). The crescendoing pattern of recruitment and contraction of rib cage muscles, abdominal muscles, and the diaphragm is similar to what occurs during pressure‐threshold loading (Whitelaw et al., 1988). This makes sense physiologically because the respiratory muscles are contracting isometrically against an infinite elastance, that is, the closed glottis.

Existing evidence suggests that apnoea divers do not have stronger‐than‐normal respiratory muscles. However, it is possible that apnoea divers have fatigue‐resistant respiratory muscles. The rationale for having fatigue‐resistant muscles would be that during competition and training, apnoea divers experience large swings in respiratory muscle pressures and, presumably, experience extreme sensations of breathlessness. Previous work, during dynamic exercise, has suggested a relationship between respiratory muscle activity and ratings of dyspnoea (Boyle et al., 2020). In this previous work, fatiguing the diaphragm prior to dynamic exercise decreased exercise performance and increased the intensity and unpleasantness of dyspnoea (Boyle et al., 2020). Thus, having fatigue‐resistant respiratory muscles could delay or minimize the presumed progressively increasing dyspnoea during a maximal apnoea, which may allow the apnoea to persist for longer during the struggle phase. However, this has yet to be tested.

Previous work supports this hypothesis via quantifying the time–tension index (TTI) of the respiratory muscles (Cross, Breskovic et al., 2013; Diniz et al., 2014) via the following equation:

$$\mathrm{TTI}=\left(\frac{P_{\mathrm{DI}}}{P_{DImax}}\right)\times \left(\frac{T_{I}}{T_{\mathrm{total}}}\right)$$

Where *P*_DI_ is the active diaphragm pressure, *P*_DImax_ is the maximal diaphragm pressure, *T*_I_ is total time of inspiration, and *T*_total_ is the time of total respiratory cycle (Cross, Breskovic et al., 2013). This index is believed to be closely related to the energetic demand of the respiratory muscles, so it provides relevant information. During a maximal breath‐hold, the time–tension index of the diaphragm has been shown to increase from ∼0.03 to ∼0.18 during the struggle phase, a 5‐ to 6‐fold increase (Cross, Breskovic et al., 2013), which is in excess of the ‘task failure’ threshold for this muscle (Bellemare & Grassino, 1982). This suggests that the diaphragm is performing work that may result in fatigue. Importantly, establishment of this task failure threshold was done via 45 min of resistive breathing whereby the diaphragm experienced far more contractions that were at or above the threshold. However, these tasks were done under normoxic and eucapnic conditions and the diver will experience significant hypoxaemia and hypercapnia (Willie et al., 2014), both of which can accelerate and exaggerate fatigue (Babcock et al., 1995; Rafferty et al., 1999; Vogiatzis et al., 2006, 2007, 2008). Nevertheless, even if task failure is met, it is possible the diaphragm will not be fatigued (McKenzie et al., 1997), but this is not known in apnoea divers following maximal breath‐holds. Similarly, the time–tension index of the rib cage muscles, taken to reflect expiratory muscle activity, increases from ∼0.02 to ∼0.30, which is a 15‐fold increase (Cross, Breskovic et al., 2013), and also beyond the ‘task failure’ threshold for these muscles (Zocchi et al., 1993). Again, this suggests that these muscles are performing fatiguing work. Clearly, the respiratory muscles are active and performing substantial work during a maximal apnoea, but they are doing so in a progressively increasing hypoxaemic and hypercapnic environment, both of which are known to lower the aforementioned ‘task failure’ thresholds (Jardim et al., 2015; Radell et al., 2000; Vogiatzis et al., 2007).

**FIGURE 3:**
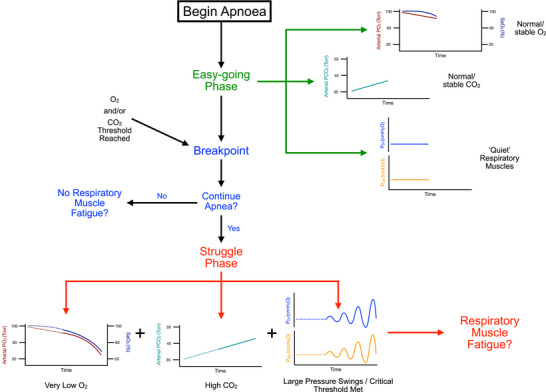
Schematic representation of a hypothesis for the presence of respiratory muscle fatigue following a maximal apnoea. During the easy‐going phase arterial blood gases are normal and respiratory muscles are not tonically active. However, arterial hypoxaemia and hypercapnia are progressively worsening over the course of the apnoea until the physiological breakpoint is reached and the struggle phase begins. During the struggle phase the respiratory muscles are involuntarily contracting against the closed glottis and the tension developed may exceed previously established thresholds for task failure. This, combined with hypoxaemia and hypercapnia, may lead to fatigue of the diaphragm and/or expiratory muscles.

Figure 3 provides a schematic representation of the possible mechanisms leading to respiratory muscle fatigue described above. The pressures generated by the respiratory muscles during the struggle phase, according to previous research, may reach a threshold for task failure (Cross, Breskovic et al., 2013). Additionally, during a maximal apnoea the diver experiences severe arterial hypoxaemia and hypercapnia (Bain et al., 2018; Willie et al., 2014). Although the duration of hypoxaemia and hypercapnia may only last for ∼30% of the apnoea, by end‐apnoea arterial blood gases are severely altered, that is, $P_{O_{2}}$ ∼20 Torr and $P_{CO_{2}}$ ∼50 Torr. Collectively, this leads to the hypothesis that respiratory muscle fatigue will be present following a maximal apnoea, but this has yet to be determined. In doing so, it may allow further exploration of the role, if any, the respiratory muscles play in the termination of a maximal apnoea via physiological or psychophysiological mechanisms, for example, severe hypoxaemia, lightheadedness or extreme breathlessness.

As discussed above, the volume of O_2_ stored in the lungs and blood likely plays a role in the duration of a maximal apnoea. The maximal capacity of the system to store oxygen is constrained, in part, by the ability for the apnoea diver to inhale as large a volume of gas as possible prior to cessation of the apnoea. This, in turn, is impacted by the ability, or rather tolerance, of the lungs and chest wall to expand during a maximal inspiration. It is, therefore, possible that elite apnoea divers have a greater lung and/or chest wall compliance compared to non‐divers. The compliance of the chest wall of a young, healthy individual can range between 134 and 334 mL/cmH_2_O (Estenne et al., 1985; Gideon et al., 2021). Chest wall compliance is considered to be linear between functional residual capacity and total lung capacity in young, healthy men and women (Estenne et al., 1985; Cross, Winters et al., 2014; Gideon et al., 2021). The static compliance of the lungs of a young, healthy individual exhibits volume dependence with compliance being the lowest near total lung capacity and relatively high near functional residual capacity. Because of the non‐linearity of lung compliance throughout the lung volume continuum, the value for lung compliance is typically taken as the slope of the pressure–volume curve 0.5 or 1.0 L above functional residual capacity. Lung compliance in healthy, young individuals can range from 150 to 400 mL/cmH_2_O (Gideon et al., 2020). Only one previous study has examined lung compliance in a group of breath‐hold divers and found it to range from 284 to 667 mL/cmH_2_O (Tetzlaff et al., 2008). They quantified static lung compliance during ‘passive, non‐interrupted expiration’, but the airflow threshold was not described. This is important because negative esophageal pressure (−*P*_ES_) is only a valid surrogate for lung recoil pressure when airflow is low enough to consider alveolar pressure to be nil (Gideon et al., 2020; Gillespie et al., 1973; Hurewitz et al., 1984). Therefore, it is not definitively known whether or not chest wall and/or lung compliance is altered in trained apnoea divers. As mentioned above, having a greater lung and/or chest wall compliance could increase apnoea duration and play a role in better understanding how elite divers can achieve such prolonged apnoeas.

## 4 WHAT REMAINS UNKNOWN ON THE RESPIRATORY RESPONSE TO MAXIMAL APNOEAS?

Above, we have outlined some of what is known regarding the respiratory responses, specifically respiratory mechanics and respiratory muscle activity, during a maximal apnoea. We also note some interesting, and potentially important, aspects that are unknown and have yet to be studied. These include strength of the respiratory muscles in elite apnoea divers. Likewise, it is unknown whether or not a maximal apnoea elicits respiratory muscle fatigue and/or if the respiratory muscles of elite apnoea divers are fatigue resistant. Finally, it is not known whether or not the compliance of the lungs and/or chest wall is altered in apnoea divers. Collectively, these may play a role, the magnitude of which is presently unknown, in maximal apnoea duration.

One interesting area of pursuit that has not been discussed above is long‐term respiratory health as a consequence of apnoea diving. In general, the assumption is that regular, routine physical activity of any kind leads to improved quality of life and longevity. However, breath‐hold divers experience repetitive periods of hypoxaemia and hypercapnia, which could be akin to sleep apnoea. One previous study has investigated the pulmonary vascular responses to alveolar hypoxia with and without sildenafil in current breath‐hold divers (Kelly et al., 2022). Divers had a blunted pulmonary vascular response to hypoxia compared to non‐divers, which suggests that there is a positive vascular adaptation, that is, no increase in pulmonary vascular pressure in response to hypoxaemia, to apnoea diving. The implication of this could be that apnoea divers are less susceptible to pulmonary hypertension and/or that apnoea training could be a therapeutic intervention of pulmonary hypertension. Importantly, whether or not this blunted response to hypoxaemia persists following cessation of apnoea diving participation is unknown. Another area worth exploring is the change in pulmonary function during the ageing process. It is a well‐known tenet of respiratory physiology that there is an age‐associated decline in respiratory function. Lifestyle choices, that is, smoking, diet, etc., are known to impact the slope of the decline in respiratory function. Some divers that dive for depth specifically experience ‘lung squeeze’. Lung squeeze is a form of barotrauma that occurs during ascent and can, in an extreme case, lead to alveolar rupture and air leaking into the peribronchial space (Mijacika & Dujic, 2016). If this occurs repeatedly it could lead to long‐term issues such as emphysema. However, this has yet to be explored.

## AUTHOR CONTRIBUTIONS

Colin D. Hubbard and Joseph W. Duke drafted the manuscript. All authors critically revised the manuscript. All authors have read and approved the final version of this manuscript and agree to be accountable for all aspects of the work in ensuring that questions related to the accuracy or integrity of any part of the work are appropriately investigated and resolved. All persons designated as authors qualify for authorship, and all those who qualify for authorship are listed.

## CONFLICT OF INTEREST

The authors have no conflicts of interest.
